# Accuracy of and preferences for blood-based versus oral-fluid-based HIV self-testing in Malawi: a cross-sectional study

**DOI:** 10.1186/s12879-024-09231-1

**Published:** 2024-04-02

**Authors:** Ailva O’Reilly, Webster Mavhu, Melissa Neuman, Moses K. Kumwenda, Cheryl C. Johnson, George Sinjani, Pitchaya Indravudh, Augustin Choko, Karin Hatzold, Elizabeth L. Corbett

**Affiliations:** 1 Malawi Liverpool Wellcome Trust, Blantyre, Malawi; 2 Centre for Sexual Health and HIV/AIDS Research (CeSHHAR) Zimbabwe, Harare, Zimbabwe; 3 Department of International Public Health, Liverpool School of Tropical Medicine, Liverpool, UK; 4 Department of Infectious Disease Epidemiology, London School of Hygiene & Tropical Medicine, London, UK; 5 Global HIV, Hepatitis and STI Programmes, World Health Organization, Geneva, Switzerland; 6 Department of Global Health and Development, London School of Hygiene & Tropical Medicine, London, UK; 7 Population Services International, Cape Town, South Africa; 8 Department of Clinical Research, London School of Hygiene & Tropical Medicine, London, UK

**Keywords:** HIV, Self-testing, Cross-sectional study, Malawi, Sub-Saharan Africa, Men

## Abstract

**Background:**

HIV self-testing (HIVST) can use either oral-fluid or blood-based tests. Studies have shown strong preferences for self-testing compared to facility-based services. Despite availability of low-cost blood-based HIVST options, to date, HIVST implementation in sub-Saharan Africa has largely been oral-fluid-based. We investigated whether users preferred blood-based (i.e. using blood sample derived from a finger prick) or oral fluid-based HIVST in rural and urban Malawi.

**Methods:**

At clinics providing HIV testing services (*n* = 2 urban; *n* = 2 rural), participants completed a semi-structured questionnaire capturing sociodemographic data before choosing to test using oral-fluid-based HVST, blood-based HIVST or provider-delivered testing. They also completed a self-administered questionnaire afterwards, followed by a confirmatory test using the national algorithm then appropriate referral. We used simple and multivariable logistic regression to identify factors associated with preference for oral-fluid or blood-based HIVST.

**Results:**

July to October 2018, *N* = 691 participants enrolled in this study. Given the choice, 98.4% (680/691) selected HIVST over provider-delivered testing. Of 680 opting for HIVST, 416 (61.2%) chose oral-fluid-based HIVST, 264 (38.8%) chose blood-based HIVST and 99.1% (674/680) reported their results appropriately. Self-testers who opted for blood-based HIVST were more likely to be male (50.3% men vs. 29.6% women, *p* < 0.001), attending an urban facility (43% urban vs. 34.6% rural, *p* = 0.025) and regular salary-earners (49.5% regular vs. 36.8% non-regular, *p* = 0.012). After adjustment, only sex was found to be associated with choice of self-test (adjusted OR 0.43 (95%CI: 0.3–0.61); *p*-value < 0.001). Among 264 reporting blood-based HIVST results, 11 (4.2%) were HIV-positive. Blood-based HIVST had sensitivity of 100% (95% CI: 71.5–100%) and specificity of 99.6% (95% CI: 97.6–100%), with 20 (7.6%) invalid results. Among 416 reporting oral-fluid-based HIVST results 18 (4.3%) were HIV-positive. Oral-fluid-based HIVST had sensitivity of 88.9% (95% CI: 65.3–98.6%) and specificity of 98.7% (95% CI: 97.1–99.6%), with no invalid results.

**Conclusions:**

Offering both blood-based and oral-fluid-based HIVST resulted in high uptake when compared directly with provider-delivered testing. Both types of self-testing achieved high accuracy among users provided with a pre-test demonstration beforehand. Policymakers and donors need to adequately plan and budget for the sensitisation and support needed to optimise the introduction of new quality-assured blood-based HIVST products.

## Background

Global (95–95-95) targets are that by 2025, 95% of all people living with the Human Immunodeficiency Virus (HIV) will know their HIV status, 95% of all people with diagnosed HIV infection are on sustained HIV treatment and, 95% of all people on treatment are virally suppressed [1]. HIV self-testing (HIVST) is recommended as a safe, acceptable and effective approach to increase access to and uptake of HIV testing [2–7].

Studies from a wide range of countries have established that HIVST is highly acceptable and accurate among various population groups [8–21]. Within sub-Saharan Africa, HIVST is becoming increasingly important in reaching the remaining 12% of people with HIV who are still unaware of their status [22]. Currently, 79% of countries in the region have HIVST policies, however only 45% of these countries are fully implementing [23]. To scale up HIVST implementation further, it is essential that quality-assured and affordable products are available alongside strengthened supply chain systems and increased user awareness [24].

Both blood-based self-testing (BBST) (i.e. using blood sample derived from a finger prick) and oral-fluid-based self-testing (OFBST) have been found to be accurate and acceptable particularly with demonstrations and training before self-testing for the first time in sub-Saharan Africa [25], and there are now five blood-based products and one oral-fluid-based product for HIVST which are prequalified by the World Health Organization [26]. There are perceived advantages and disadvantages of either method. For example, OFBST is widely viewed as simple, painless, quick and less invasive [27, 28], but characterised by lower sensitivity [12, 24]. BBST is perceived as more accurate [27] and having a higher sensitivity [24], but more invasive and complex to perform [29]. Despite the availability of several different (blood-based) products, some of which cost as low as $1.00, most HIVST implementation to date has been oral-fluid-based owing to the simple nature of the oral fluid-based HIVST kits, despite their higher minimum cost of $2.50. Wide-scale implementation of blood-based HIVST in sub-Saharan Africa is hampered by many bottlenecks including lack of quality-assured products, supply chain issues and lack of awareness among potential users [15, 19].

Here, we report on a cross-sectional study investigating preference for oral-fluid or blood-based HIVST and the accuracy of HIVST results, based on the results which untrained participants reported after conducting their own testing, in a real-world setting. We also explored reasons for accepting HIVST and participants’ preferences for oral-fluid and blood-based HIVST among people attending rural and urban facilities in Malawi.

## Methods

### Study design, setting and participants

We conducted the cross-sectional study at four facilities (*n* = 2 urban; *n* = 2 rural) offering HIV testing services in Blantyre, Malawi. Participants were eligible for the study if they were ≥ 16 years old and not already aware to be HIV positive.

### Study procedures

Clients seeking HIV testing services at the four facilities were approached by the facility healthcare provider and, after consenting to participate, directed to the study counsellor. Consenting participants completed a short interviewer-administered questionnaire which captured sociodemographic data. Participants then chose one of three HIV testing options: oral-fluid-based HIVST, blood-based HIVST or provider-delivered blood-based testing. Regardless of choice of testing method, all participants received testing within the health facility of their original attendance, where they either self-tested in a private space or, if they chose provider-delivered testing, were tested by a HIV provider with no additional study follow-up.

Those that opted for self-testing (either type) were asked to select a statement that best described their reason for choosing that testing modality. Each participant was then shown a brief pre-test demonstration specific to the self-test type they had chosen (BBST ~ 5–8 min, OFBST ~ 2 min). Participants were also provided with written and pictorial instructions which they could check whilst self-testing. After self-testing without direct assistance from a provider, individuals then completed a simple form on which they ticked one of three options: HIV negative, HIV positive or not sure/invalid. The used self-test device was then collected by a health worker and the result re-read within 40 min of self-testing by the participant. The health worker recorded the ‘re-read result’ whilst unaware of the result which the participant had recorded. Options here differed slightly and were: negative, faint positive, clear positive and invalid.

For those clients who self-tested, the health worker performed confirmatory testing using both Determine and Unigold finger-prick rapid tests (national testing algorithm in Malawi) in parallel and recorded the results. Afterwards, they administered a questionnaire which asked participants about both their experience and perspective of HIVST. Appropriate referral was carried out by the health worker for any positive results with HIVST.

#### Testing methods

Self-testing used OraQuick HIV Self-test (OraSure Technologies Inc., Bethlehem, PA, USA) run using oral-fluid specimens and INSTI HIV Self-test (BioLytical® Laboratories Inc., Richmond, B.C., Canada) run from finger-prick specimens. For reference, the manufacturer-reported sensitivity and specificity are 99.3% and 99.8%, respectively for the OraQuick test and 99.8% and 99.5% for INSTI (using finger-prick blood) (www.fda.gov).

Corresponding with the concurrent national HIV testing algorithm in Malawi at the time, Determine (Abbott Laboratories) and Unigold (Trinity Biotech plc) rapid diagnostic tests were also performed on finger-prick blood.

#### Outcomes and measurements

##### Accuracy of self-read HIV self-test results

The main outcomes were the sensitivity and specificity of blood-based and oral-fluid-based self-tests, with respect to the participant-reported result. Accuracy (sensitivity and specificity) was assessed by comparing results with (a gold standard of) the national testing algorithm in Malawi (Determine and Unigold). If required, participants received further testing at the health facility of their original attendance in accordance with the national algorithm for discrepant results.

##### Sociodemographic characteristics

The interviewer selected either ‘Yes,’ ‘No’ or one option from a list in answer to questions about each participant’s sociodemographic status.

##### Reasons for opting for self-test type

Clients selected a statement that best described their reason for choosing that testing modality.

##### Post-test questionnaire outcomes

Clients’ experience of HIVST: Clients selected from lists of options to describe who was present when they self-tested, whether and to whom they disclosed their result, whether they had any regrets about self-testing and what actions they took afterwards.

Clients’ perspective on HIVST: Clients selected from lists of options to describe how confident they were that their result was accurate and which combination of test type and setting they would prefer for their next HIV test.

#### Statistical methods/analyses

Data were stored in a SQL database, encoded and analysed using Stata version 16 (College Station, Texas, USA). Only participants who proceeded with HIV testing were included in the analysis. Participants who selected the incorrect self-test type when asked which they had used were excluded from the post-test questionnaire analysis.

##### Descriptive analysis

Using data from the sociodemographic questionnaire, variables describing sociodemographic characteristics were cross-tabulated by chosen HIV test type and column percentages calculated. For comparisons of categorical and continuous data, percentages and means were used, respectively. Missing data were evaluated for each variable by computing the proportion missing, with the denominator as the total number of participants who agreed to test, and the numerator being the total with a missing response.

To describe the reasons why clients opted for a test type, the proportions of clients selecting each particular reason from the list of available options were estimated. Proportions were compared as percentages by self-test type.

##### Analysis of factors affecting self-test choice

A priori, the intended aim was specifically to compare factors associated with selecting blood-based versus oral-fluid-based self-test rather than provider-delivered blood-based testing versus (any) self-testing. Therefore, participants who opted for provider-delivered testing were excluded from this analysis. Proportions of participants selecting each self-test type (OFBST vs. BBST) were compared by each sociodemographic variable and, for the crude analysis, p-values were obtained by Χ^2^ tests for all variables except age (t-test). *P*-values of < 0.05 were regarded as significant. There was no adjustment for potential confounders in this analysis.

Analysis was also performed using multivariable logistic regression to estimate adjusted odds ratios and *p*-values for the relationship between choice of self-test type and selected variables. Variables were included as potential confounders in the adjusted model if they had been found to be associated with self-test choice based on Χ^2^ testing or if they were factors often stated in the literature to be associated. On this basis, we adjusted for age, sex, literacy, location, marital status and regular income.

##### Accuracy analysis

Sensitivity and specificity for each self-test type were estimated omitting any results reported as ‘not sure/invalid’ and reported as percentages (with 95% confidence intervals, CI). These were estimated using the ‘diagti’ command in Stata by inputting the numbers of true positives, false positives, true negatives and false negatives. For any false positives or false negatives, the self-read result was compared to the re-read result by the study health worker to determine whether these were reading errors or truly inaccurate test results.

##### Analysis of post-test questionnaire responses

For the post-test survey data, we conducted a descriptive analysis. Here we simply list proportions of users selecting each category in answer to questions on their experience of HIV self-testing and their perspective on it.

## Results

From July to October 2018, approximately 1,100 clients seeking HIV testing services at the four facilities were approached by the facility service provider, who then screened for eligibility and took consent. Of these, 709/1,100 (64% response rate) were eligible and initially consented to participate. All 709 completed the sociodemographic questionnaire, of which 18/709 (2.5%) answered the questions but then opted out of any type of HIV testing and so were excluded from the analysis. There were no missing data for any sociodemographic variables.

**Fig. 1:**
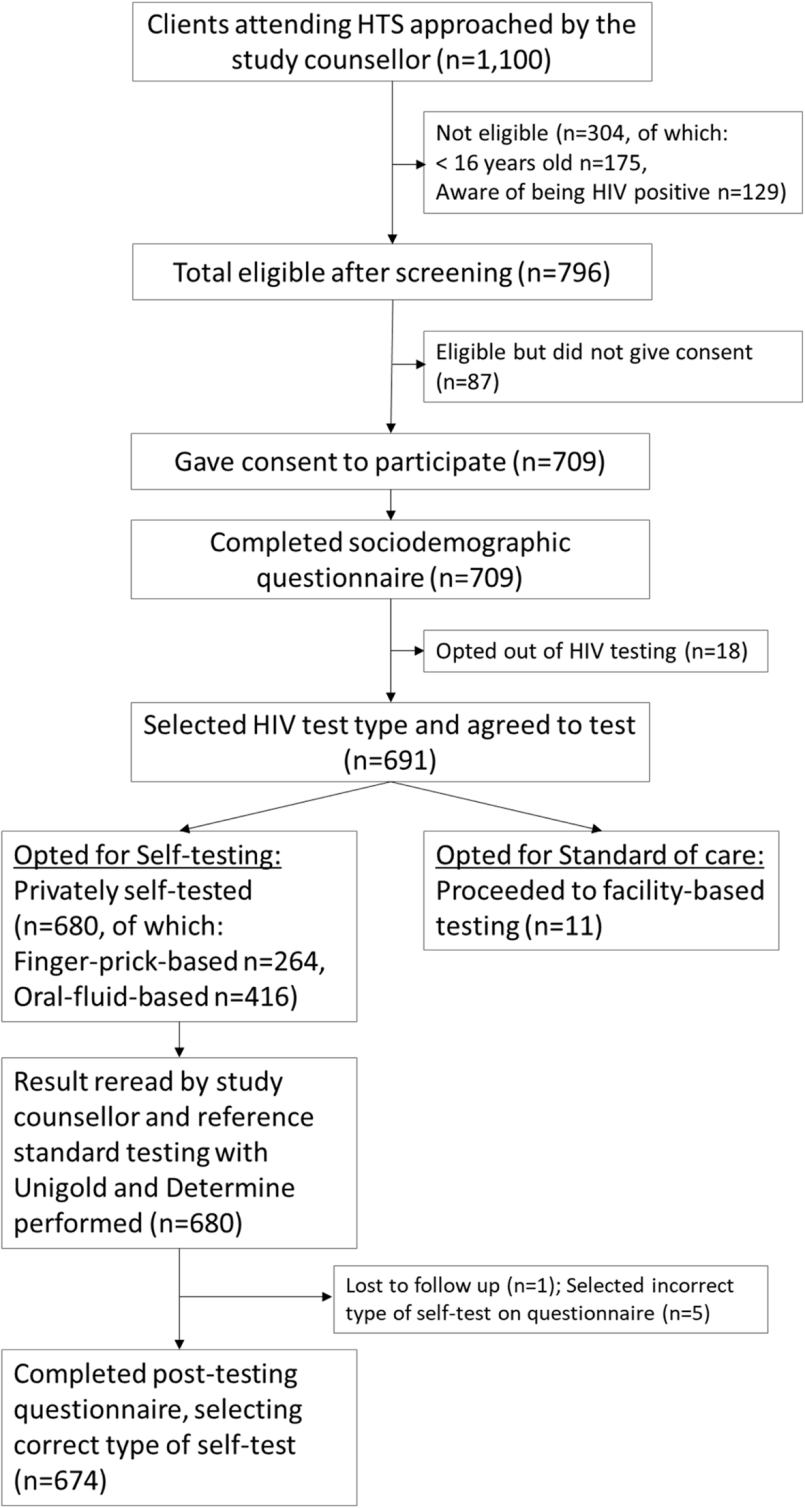
Study flow diagram

Of the remaining 691 who both completed the sociodemographic questionnaire and tested for HIV (any type), 680/691 (98.4%) opted for self-testing and only 11/691 (1.6%) opted not to self-test and therefore received only provider-delivered testing as standard of care. Of the 680 who self-tested, 679 (99.9%) completed the post-test questionnaire (1 was lost to follow-up). Of the 679, five (0.7%) mistakenly selected the wrong self-test type (2 who had used OFBST selected BBST; vice versa for 3 others). Therefore, these five responses were excluded from the post-test questionnaire analysis hence the final figure of 674 participants (*n* = 414 OFBST; *n* = 260 BBST) (Fig. 1).

Relatively equal numbers 349 (50.1%) and 342 (49.5%) were attending urban and rural health-care facilities, respectively. Over half (56.0%) were female, 52.1% were married and 45.4% were < 25 years. The overall mean age was 26.2 years (standard deviation 8.4). More than half (54.3%) had some secondary education or higher, 42.0% had some primary schooling and 3.8% had no formal schooling, with a majority of all participants (88.1%) reporting that they were literate (could read a one-page letter or newspaper in English or Chichewa). Overall, most (83.9%) did not receive a regular salary and again, most (87.4%) rated their own health to be at least good (Table 1).

**Table 1:** Sociodemographic characteristics, overall and by type of HIV test (*N* = 691)

**Characteristic**	**Type of HIV Test**			**Total (*n* = 691)**
	**Oral-fluid-based HIVST (*n* = 416)**	**Blood-based HIVST (*n* = 264)**	**Provider-delivered blood-based testing (*n* = 11)**	
	**n (%)**	**n (%)**	**n (%)**	**n (%)**
**Sex**				
Male	150 (36.1)	152 (57.6)	2 (18.2)	304 (44)
Female	266 (63.9)	112 (42.4)	9 (81.8)	387 (56)
**Age (years)**				
16–19	63 (15.1)	48 (18.2)	0 (0)	111 (16.1)
20–24	169 (40.6)	94 (35.6)	3 (27.3)	266 (38.5)
25–40	156 (37.5)	106 (40.2)	7 (63.6)	269 (38.9)
> 40	28 (6.7)	16 (6.1)	1 (9.1)	45 (6.5)
**Participants**				
Urban	195 (46.9)	147 (55.7)	7 (63.6)	349 (50.5)
Rural	221 (53.1)	117 (44.3)	4 (36.4)	342 (49.5)
**Marital status**				
Married or living as married	215 (51.7)	136 (51.5)	9 (81.8)	360 (52.1)
Never married	175 (42.1)	116 (43.9)	1 (9.1)	292 (42.3)
Widowed/Separated/Divorced	26 (6.3)	12 (4.5)	1 (9.1)	39 (5.6)
**Education attainment**				
No formal schooling	14 (3.4)	10 (3.8)	2 (18.2)	26 (3.8)
Primary	184 (44.2)	99 (37.5)	7 (63.6)	290 (42)
Secondary and above	218 (52.4)	155 (58.7)	2 (18.2)	375 (54.3)
**Literacy (Can read a newspaper or 1 page letter)**				
Yes	360 (86.5)	240 (90.9)	9 (81.8)	609 (88.1)
No	56 (13.5)	24 (9.1)	2 (18.2)	82 (11.9)
**Receive regular salary**				
Yes	55 (13.2)	54 (20.5)	2 (18.2)	111 (16.1)
No	361 (86.8)	210 (79.5)	9 (81.8)	580 (83.9)
**Self-rating of general health**				
Very good	153 (36.8)	105 (39.8)	2 (18.2)	260 (37.6)
Good	218 (52.4)	119 (45.1)	7 (63.6)	344 (49.8)
Fair to Poor	45 (10.8)	40 (15.2)	2 (18.2)	87 (12.6)

### Factors associated with choice of self-test type

Of the 680 participants opting for HIV self-testing, 416/680 (61.2%) chose oral-fluid-based HIVST and 264/680 (38.8%) chose blood-based HIVST. Table 2 shows the proportions of participants selecting each self-test type compared by various variables. Most women (266/378; 70.4%) opted for oral-fluid-based HIVST whilst about half of men (150/302; 49.7%) opted for this testing modality (*p* < 0.001). Participants testing in rural facilities frequently opted for oral-fluid-based HIVST (221/338; 65.4%) whilst those testing in urban facilities frequently chose blood-based HIVST (147/342; 43.0%) (*p* = 0.025). A greater proportion of illiterate participants opted for oral-fluid-based HIVST (70.0%; 56/80) than those who were literate (60.0%; 360/600) although this difference was not statistically significant *p* = 0.085.

**Table 2:** Factors associated with choice of self-test type (unadjusted analysis)

**Variable**	**Type of HIV Self-Test**				*p* -value*
	**Blood-based**		**Oral-fluid-based**		
	N	%	N	%	
**Sex**					
Male (*n* = 302)	152	50.3	150	49.7	< 0.001
Female (*n* = 378)	112	29.6	266	70.4	
**Age**					
Mean (SD)	25.8	7.7	26.3	8.9	0.455
**Age group**					
16–19 (*n* = 111)	48	43.2	63	56.8	0.499
20–24 (*n* = 263)	94	35.7	169	64.3	
25–39 (*n* = 262)	106	40.5	156	59.5	
> = 40 (*n* = 44)	16	36.4	28	63.6	
**Location of primary healthcare facility**					
Urban (*n* = 342)	147	43.0	195	57.0	0.025
Rural (*n* = 338)	117	34.6	221	65.4	
**Educational attainment**					
No formal schooling (*n* = 24)	10	41.7	14	58.3	0.222
Primary (*n* = 283)	99	35.0	184	65.0	
Secondary and above (*n* = 373)	155	41.6	218	58.4	
**Literate? (Can read a newspaper or letter)**					
Yes (*n* = 600)	240	40.0	360	60.0	0.085
No (*n* = 80)	24	30.0	56	70.0	
**Receiving a regular salary**					
Yes (*n* = 109)	54	49.5	55	50.5	0.012
No (*n* = 571)	210	36.8	361	63.2	
**Self-rating of general health**					
Very good (*n* = 258)	105	40.7	153	59.3	0.102
Good (*n* = 337)	119	35.3	218	64.7	
Fair to Poor (*n* = 85)	40	47.1	45	52.9	
**Marital status**					
Married or living as married (*n* = 351)	136	38.7	215	61.3	0.615
Never married (*n* = 291)	116	39.9	175	60.1	
Widowed/Separated/Divorced (*n* = 38)	12	31.6	26	68.4	

^*^ Obtained using Χ^2^ for all variables except age; obtained using t-test for age

There was some evidence of an association between the receipt of a regular salary and choice of test; a greater proportion of participants not receiving a regular salary chose oral-fluid-based HIVST (63.2%; 361/571) compared to those with a regular salary (50.5%; 55/109) (*p* = 0.012). In the unadjusted analysis, type of self-test selected was not found to be associated with any of the remaining variables (Table 2).

### Accuracy analysis

HIV prevalence among those participants who opted for self-testing was 4.3% (29/680) (Table 3).

**Table 3:** Accuracy of self-read HIV self-tests against gold standard^a^

**Self-test Type**	**True Positives n (%)**	**False Negatives n (%)**	**False Positives n (%)**	**True Negatives n (%)**	**Sensitivity (95% CI)**	**Specificity (95% CI)**
Oral-fluid-based (*n* = 416)	16 (3.8)	2 (0.5)	5 (1.2)	393 (94.5)	88.9% (65.3–98.6%)	98.7% (97.1–99.6%)
Blood-based (*n* = 244^b^)	11 (4.6)	0 (0)	1 (0.4)	232 (95.1)	100.0% (71.5–100.0%)	99.6% (97.6–100.0%)

No results were excluded for oral-fluid-based HIVST

^a^The gold standard is based on the result of two confirmatory tests – Unigold and Determine rapid tests. There were no discordant results (100% concordance of Unigold and Determine tests)

^b^20 of the 264 blood-based test results were excluded from the accuracy calculations. For each of these 20 results, participants had recorded ‘not sure/invalid’ as the result

Based on participant-reported results, the sensitivity of oral-fluid-based HIVST was 88.9% (16/18; 95% CI: 65.3–98.6%) and the specificity was 98.7% (393/398; 95% CI: 97.1–99.6%). No participants recorded ‘not sure/invalid’ with this test. There were five oral-fluid-based HIVST false positives, four of which the health worker had recorded on re-read as ‘faint positive’ and one of which was a reading error (recorded as negative on re-read). There were two oral-fluid-based HIVST false negatives, for which the re-read result agreed (also recorded as negative on re-read) (Table 3).

Based on participant-reported results, the sensitivity of blood-based HIVST was 100.0% (11/11; 95% CI: 71.5–100.0%) and the specificity was 99.6% (232/233; 95% CI: 97.6–100.0%). Twenty (7.6%; 20/264) results had been recorded as ‘not sure/invalid’ and were excluded from this calculation. For all of these, the re-read by the health worker was recorded as ‘invalid.’ The ‘gold-standard’ result for all these 20 was negative. The one false positive blood-based HIVST result was a reading error (recorded as negative on re-read) (Table 3).

### Reasons for opting for self-testing type

Of the 416 clients who performed oral-fluid-based HIVST, data were missing for 12 (2.9%). Of the 264 who performed blood-based HIVST, data were missing for two (0.8%) for this part of the questionnaire. Among those who chose oral-fluid-based HIVST, the majority (76.5%; 309/404) reported that they had done so because it was ‘easier to use than the blood-based self-test’. Among those who chose blood-based HIVST, the most frequently selected reasons for doing so were ‘interested in new technologies’ (32.1%; 84/262) and ‘more accurate than the oral-fluid-based self-test’ (30.9%; 81/262) (Table 4).

**Table 4:** Reasons for opting for self-test type

**Reason**	**Test type**	
	OFBST (*N* = 404) n ( %)	BBST (*N* = 262) n (%)
Curious about this specific self-test kit	29 (7.2)	26 (9.9)
Want to be the first to know my result	6 (1.5)	8 (3.1)
Like the opportunity to test in private	24 (5.9)	28 (10.7)
Interested in new technologies	30 (7.4)	84 (32.1)
It is something I could do again and wanted to try it out	2 (0.5)	0 (0)
Easier to use than the other self-test offered	309 (76.5)	32 (12.2)
More accurate than the other self-test offered	0 (0)	81 (30.9)
Other reason	4 (1.0)	1 (0.4)

### Experiences and perspectives of self-testing – post-test survey

After excluding five participants who selected the incorrect self-test type (i.e. they ticked that they had done OFBST when in fact they had done BBST or vice versa), the analysis of questionnaire answers was conducted among 674 participants (414 OFBST; 260 BBST). Among these, 418/674 (62.0%) were alone when they tested and 256/674 (38.0%) had somebody else with them: 154/256 (60.2%) had a spouse/partner, 50/256 (19.5%) had another family member, 47/256 (18.4%) had a friend, 4/256 (1.6%) had a healthcare worker and one (0.4%) participant answered 'other'. 60.4% (407/674) reported disclosing their result to someone. Of these, 43.5% (177/407) disclosed to their partner/spouse. The remainder selected 'other' (28.0%; 114/407), 'other family member' (21.4%; 87/407), parent (6.4%; 26/407) or employer (0.7%; 3/407). Only a minority (3.0%; 20/674) felt regret having taken the test when asked to look back at it. Only three (0.4%) participants felt they were pressured to disclose their result to another person, one by a partner/spouse, one by a parent and one by 'other.'

In response to the question ‘What actions did you take after your last HIV test?’ 416/674 (68.4%) stated that they confirmed their self-test result, 11 (1.6%) indicated they went for HIV care (although only nine of these had tested positive on both self-test and Unigold/Determine), 88 (13.1%) got condoms and 123 (18.3%) reported that they ‘did not do anything.’ Of the 300 men included in the questionnaire, 54 (18.0%) reported deciding that they would like to have voluntary medical male circumcision (VMMC).

Most (90.2%; 608/674) reported that they were ‘very confident’ that their test result was accurate. The remainder were ‘somewhat confident’ (57/674; 8.5%) or ‘not very confident’ (9/674; 1.3%). From the available options for combination of test type and setting (in response to the question ‘Which would you most want to be your next HIV test?’), participants most frequently selected the option ‘blood-based self-testing *without* a counsellor present’ (243/674; 36.1%). The second most popular option was ‘oral-fluid-based self-testing *without* a counsellor present’ (236/674; 35.0%), followed by ‘oral-fluid-based self-testing *with* a counsellor present’ (141/674; 20.9%) in third place. The least popular options included ‘blood-based self-testing *with* a counsellor present’ (34/674; 5.0%), ‘testing by a counsellor at a hospital, clinic or health centre’ (19/674; 2.8%) and ‘testing by a counsellor at a mobile clinic’ (1/674; 0.2%). None selected the option ‘testing by a counsellor at home.’

## Discussion

We conducted a cross-sectional study to investigate the preference for blood-based versus oral fluid-based HIV self-testing among lay users attending rural and urban health facilities in Malawi. We also explored reasons for accepting self-testing and participants’ preferences for oral-fluid and blood-based HIVST. Overall, we found that with minimal pre-test demonstration, individuals could accurately self-test with either oral-fluid-based or blood-based kits. While preferences for both blood-based and oral-fluid-based HIVST were high compared to the traditional provider-delivered testing, oral-fluid-based HIVST was most preferred overall. These findings can inform future HIVST implementation and procurement planning.

Accuracy of participant-reported results was acceptable for both HIVST options and similar to previous studies [25], but highest for blood-based HIVST, which achieved 100% sensitivity and 100% specificity. The lower sensitivity of oral-fluid-based HIVST observed may have been driven by retesting among people on antiretroviral therapy, which can sometimes lead to false negative results [30, 31], but this was not fully assessed in this study. Despite otherwise good performance, users of blood-based HIVST had a higher rate of individuals reporting invalid results when compared with users of oral-fluid-based HIVST (7.6% vs. 0%). The exact causes of invalid results reported were unclear, but a previous systematic review found invalid results with blood-based HIVST were slightly higher than with oral-fluid-based HIVST [25]. Some of the key challenges were due to difficulty with sample collection and transfer steps. Further, since we observed that providers also made errors when reading blood-based HIVST results, it is possible that overall, individuals—both clients and providers, were less familiar with these new test kits and more training and demonstration was needed. Since we did not conduct further testing on inconclusive or discrepant results, we could not rule out that individuals with known HIV status on antiretroviral therapy could have been included.

At the time of the study, providers and self-testers were likely to have been much more familiar with oral-fluid-based HIVST, which has been implemented in Malawi since 2011. Early studies with oral-fluid-based HIVST did report higher rates of invalids, such as in Kenya [12], where initial implementation resulted in 14.3% invalid results. However, this resolved as users became more familiar with oral-fluid-based HIVST. Therefore, perhaps one way to reduce the numbers of invalid results with blood-based HIVST might be to provide additional support and training during early-stage implementation and then scale down over time.

This study adds to the growing literature around evaluations of preferences for HIV testing in sub-Saharan Africa. When given a choice between blood-based and oral fluid-based HIVST, 61% and 39% opted for oral-fluid-based HIVST compared to blood-based self-testing, respectively. An observational study conducted in Zimbabwe among different populations also found high preference of oral-fluid-based to blood-based HIVST (50% vs. 35%, respectively) [29]. Collectively, these findings buttress recommendations to increase choice of testing modalities to enhance HIV testing acceptability and uptake [32–34]. Importantly, men (hard-to-reach) were more likely to opt for blood-based HIVST. Considering the need to prioritise testing in this group [29, 35], it appears important to offer this option to promote greater access to and uptake of testing, which is essential to achieve global testing and treatment targets [22].

Increasing preference for oral-fluid-based to blood-based HIVST is linked to perceptions of the two procedures. In this study, over three-quarters (77%) thought oral-fluid-based HIVST was easier to conduct. This concurs with findings from the Zimbabwean study where participants perceived oral-fluid-based HIVST to be both less invasive and painless [29]. An interesting pattern continues to be observed among those that choose blood-based HIVST over oral-fluid-based HIVST. As found elsewhere [29], we found that (in Malawi) these participants are more likely to be urban, literate and earning a regular salary, with their main motivation being “to try new technologies”. As different HIV testing approaches are preferred by different groups, this highlights once again, the need to provide more choices [32–34].

Of note, doubts over the accuracy of oral-fluid-based HIVST found in this and other studies [27, 29], need to be appropriately addressed to enhance acceptability and uptake of this testing modality.

Findings on preferences for the “next test” are likely to be helpful in informing scale-up of HIVST going forward. The least preferred options included any testing involving a provider, regardless of venue. Men are especially concerned about being tested at a facility and deductive disclosure occurring in the event they are positive [36, 37]. HIVST provision should therefore be sensitive to various groups and individuals’ concerns and continue to ensure privacy [35, 38]. Following testing, some participants reportedly linked to post-test services (VMMC and HIV care), highlighting once again how testing is an entry point into HIV prevention, treatment, care and support services [4, 39, 40].

A strength of this cross-sectional study is that it explored preferences for several HIV testing modalities and among different settings. Also, we had equal numbers attending urban and rural health-care facilities. The study therefore provided a balanced range of perspectives. A potential limitation is that we did not explore in-depth some of the study findings (e.g. qualitatively), which would have provided context and nuances. Additionally, we made a conservative estimate (N = 1100) of the number of people who may have presented to the health facilities from which we recruited. Therefore, it is possible that the true number of eligible people may exceed the 709 that we reported here but the results are representative of the patient population. It is worth pointing out that this response rate of 64% may be considered low.

Lastly, many factors may motivate choice and preference. Therefore, choice at one point in time does not necessarily reflect overall preference, especially among those who have not been exposed to all options available [29].

## Conclusions

This cross-sectional study enabled us to explore the accuracy of and preferences for oral-fluid vs. blood-based HIVST. Both types of self-testing achieved acceptable levels of accuracy and blood-based achieved almost complete accuracy. Both oral-fluid and blood-based HIVST are preferred over provider-delivered testing. Patterns of preference were observed whereby women, those not receiving a regular salary and participants attending rural facilities were more likely to opt for oral-fluid-based HIVST. Such information is helpful for HIVST programme policy makers who should be aware of specific groups’ behaviours/preferences when planning procurement at the population level.

Efforts to optimise implementation of blood-based HIVST are needed. Additional support and training may help to achieve optimal performance, especially during the initial stages of implementation, in which efforts to expedite familiarity with the procedure may help to reduce the frequency of invalid results more rapidly. Study findings are relevant to all programmes, ministries and implementers involved in scaling up HIVST.

## Data Availability

The datasets used and/or analysed during the current study are available from the corresponding author on reasonable request. HIV self-testing data cannot be shared.

## Abbreviations

BBST: Blood-based self-testing
CI: Confidence Interval
HIV: Human Immunodeficiency Virus
HIVST: HIV self-testing
OFBST: Oral-fluid-based self-testing
SQL: Structured Query Language
VMMC: Voluntary medical male circumcision
